# Digitalized mass distribution campaign of insecticide-treated nets (ITNs) in the particular context of Covid-19 pandemic in Benin: challenges and lessons learned

**DOI:** 10.1186/s12936-020-03508-x

**Published:** 2020-11-25

**Authors:** Rock Aïkpon, Cyriaque Affoukou, Benjamin Hounpkatin, Dieu-Donné Eclou, Yves Cyaka, Elijah Egwu, Narcisse Agbessi, Filémon Tokponnon, Sahidou Salifou, Lamidhi Salami, Aurore Ogouyemi Hounto

**Affiliations:** 1 Programme Nationale de Lutte Contre Le Paludisme (PNLP), Cotonou, Benin; 2 Ministère de La Santé, Cotonou, Benin; 3 Instance Nationale de Coordination (INC), Cotonou, Benin; 4 Alliance for Malaria Prevention, Geneva, Switzerland; 5 Catholic Relief Services, Cotonou, Benin; 6 Centre de Recherche Entomologique de Cotonou (CREC), Cotonou, Benin; 7 Université D’Abomey-Calavi, Cotonou, Benin

**Keywords:** Digital, Itns campaign, Covid-19, Lessons, Benin

## Abstract

**Background:**

In 2020, Benin has implemented a digitalized mass distribution campaign of insecticide-treated nets (ITNs) in the particular context of COVID-19 pandemic. This paper describes the implementation process as well as the challenges and lessons learned from this campaign.

**Methods:**

A descriptive design was used for reporting the planning and implementation process of ITNs campaign. Moreover, the changes and adaptations related to COVID-19 pandemic are described.

**Results:**

A total of 3,175,773 households were registered corresponding to a total of 14,423,998 persons (13.55% more from projection). Moreover, 94.16% (13,581,637 people) of enumerated population were protected. A total of 7,652,166 ITNs were distributed countrywide.

**Conclusions:**

High political commitment, engagement and support add to the financial and technical supports from partners were the essential factors that make 2020 ITNs mass campaign success in Benin despite the particular context of COVID-19 pandemic. It is essential to maintain the prevention activities for malaria and this could substantially reduce the overall impact of the COVID-19 pandemic for the populations at malaria risk.

## Background

Malaria remains endemic and a serious threat to development in inter-tropic countries, with an estimated 228 million cases and 405,000 deaths in 2018, of which 93% of cases and 94% of deaths occurred in the Africa region [1].

Vector control is a key component in malaria prevention strategies and has contributed to a significant decrease in malaria worldwide [2–4]. Insecticide-treated nets (ITNs) remain one of the most efficacious vector control measure available against malaria [5, 6] and its use has highly increased in sub-Saharan Africa in the past decade. The World Health Organization (WHO) recommends universal coverage in the populations at risk through mass distribution campaigns (with one net for every two people) [7]. The big challenge to the National Malaria Control Programme (NMCPs) is to reach and sustain this high coverage rate.

The NMCP of Benin has adopted a policy of mass distribution since 2011, on a triennial basis, considering the lifespan of ITNs, as previously evaluated in the country [8], and as recommended by the WHO [9–12]. The 4th mass distribution campaign took place in 2020 and has been digitized. The purpose of using digital tools for the 2020 ITN campaign was to collect more accurate data on the size of the population, and evaluate the speed in data collection during the campaign, as far as household enumeration and ITNs distribution phases are concerned.

In addition, during the campaign process, between the enumeration phase and that of the distribution itself, the COVID-19 pandemic occurred and Benin also recorded its first cases. Recognizing the heavy toll that malaria exacts on vulnerable populations in Africa region, the WHO recommended continuing with the implementation of malaria control interventions, such as ITNs and indoor residual spraying campaign. On this basis, the Government of Benin, through the Ministry of Health and the NMCP, decided to continue with the implementation of the distribution campaign. For this, it was necessary to revise the initial distribution protocol and take precautionary measures in order to minimize the risk of transmission of COVID-19 during distribution.

This report describes the implementation process of the 2020 ITN mass distribution campaign in Benin. The specific objectives are: (i) to describe the planning process; (ii) to describe the changes and adaptations that occurred during the distribution, due to the occurrence of the COVID-19 pandemic; (iii) to share outcome, challenges and lessons learned from the mass distribution campaign.

## Methods

### Context

**Fig. 1:**
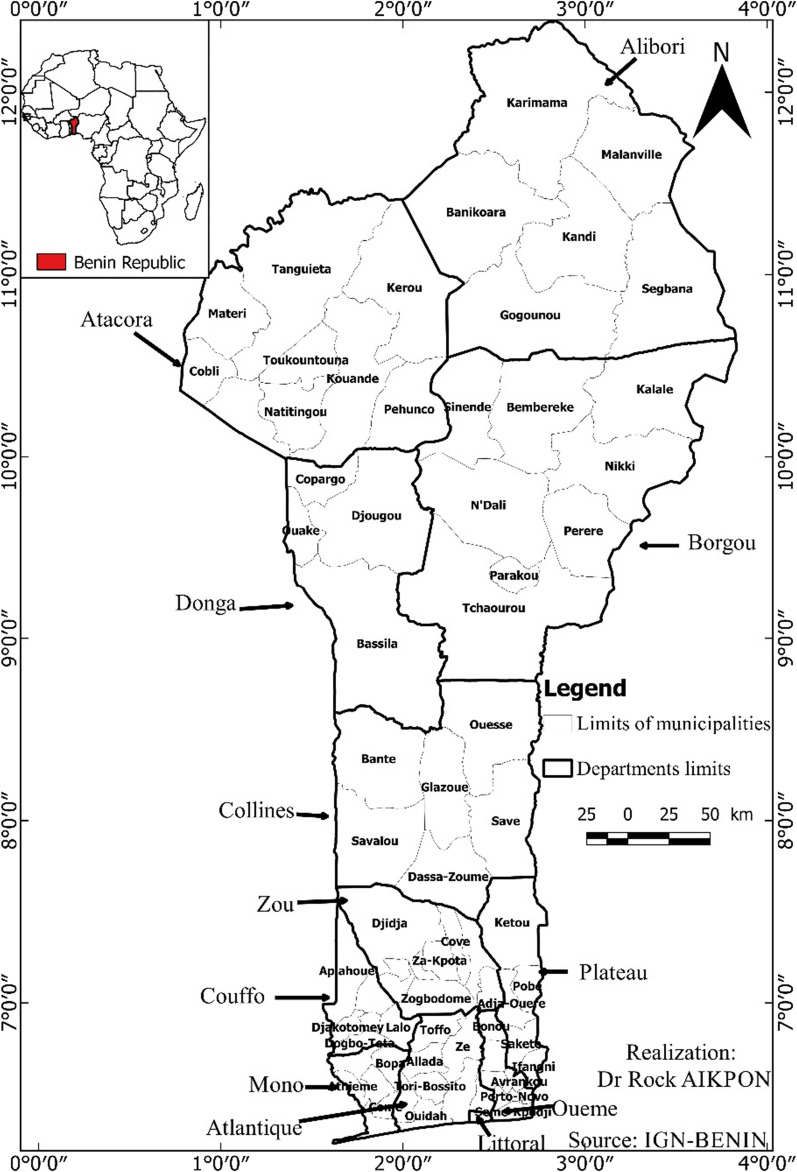
Map of Benin

Benin’s population is estimated at 12,114,193 inhabitants in 2020 [13]. The country is divided into 77 communes grouped into 34 health zones and 12 departments (Fig. 1). Malaria is endemic in all parts of country with seasonal variations. All of Benin’s population is at risk of malaria infection, which is the leading cause of morbidity and mortality. The incidence of the disease in 2018 was 18.5% in the general population with 1,755,597 confirmed cases of malaria in public health facilities and almost 2,251 deaths due to malaria, most of which are in children under 5 years [14].

As far as the evolution of the COVID-19 pandemic is concerned, Benin recorded its first official case on March 19, 2020 after the enumeration phase. On the eve of the distribution phase, there were 26 official cases with one death. Just after the distribution phase, 64 cases and one death were officially reported. As of November 05, 2020, there are a total of 2781 cases and 43 deaths.

### Digital aspect of the ITNs mass campaign

Catholic Relief Services (CRS) gave support to the Government of Benin and the NMCP in digitizing the 2020 ITNs mass campaign. CRS commenced its work with planning and capacity building sessions with NMCP staff. There was training of campaign staff on the use of digital tools and over 27,000 participants at these sessions were digitally-tracked using their biometrics as a mean of validating their attendance to each training session. The digital platform is the Cash-and-Asset Transfer Platform (CAT). A total of 3,382 smartphones and 350 solar chargers were used to perform a household enumeration to register households within the country to obtain a robust population database. It consists during the enumeration phase to collect using smartphones, household information (size, name, gender and age of household members), and then a coupon was assigned to the head of household with a unique Quick Response (QR) Code. The same digital platform was used to track distribution of ITNs and verify that all households registered received the correct number of ITNs allocated to them. The data collected on CAT was available through an online dashboard, updated in real time, allowing field supervisors to make important decisions effectively, and efficiently tracking household coverage rates as households missed were identified using sequencing and geospatial analytical dashboards easily accessible for field supervisors.

### ITNs campaign implementation process

**Fig. 2:**
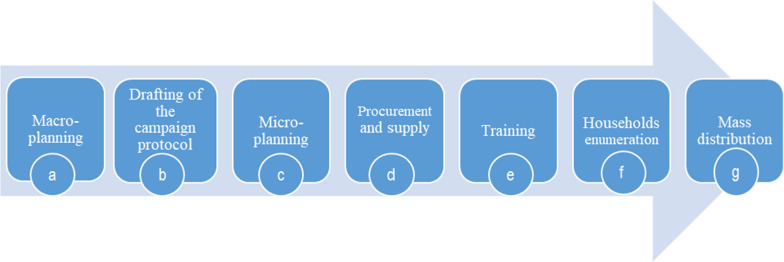
ITNs mass distribution implementation process

Figure 2 shows the different sections and the implementation process of ITNs mass distribution campaign.

#### Macro-planning

Macro-planning consisted in quantifying the number of ITNs required to achieve universal coverage according to the 2020 population projection data. In addition, the strategies for the household enumeration as well as for the ITNs distribution were designed.

#### Drafting of the campaign protocol

After the macro, the protocol of the campaign was written in great detail. This protocol was then validated by all stakeholders and consisted henceforth the campaign roadmap.

#### Micro-planning

A comprehensive micro-plan for the 2020 mass distribution was developed, which contains a rigorous gap analysis and the procurement plans as well as all details on campaign processes and a roadmap. The roles and responsibilities of actors at different levels of the health pyramid (central, department, health zone (HZ), and commune) have been clearly described.

#### Procurement and supply

A total of 8,609,873 ITNs were procured for this campaign for the whole country. All the ITNs used for the distribution campaign were manufactured in Asia (China and Vietnam). These ITNs were acquired by the government of Benin with the support of financial partners, including the Global Fund, the Bill & Melinda Gates Foundation and the United States Agency for International Development (USAID) through the President Malaria Initiative (PMI). ITNs were received in-country (without passing through central storage) and transported as planned from central level to department level and then to sub-division level. From there, they were transported to village level storage.

At the department warehouses, quality assurance was conducted prior to distribution. Samples of ITNs from different batches were sent to Centre de Recherche Entomologique de Cotonou, for physical and chemical analyses, such as stress analysis, insecticide content, fabric weight, netting, mesh size. A total of 837 ITNs were tested. All the samples conformed to the WHO procurement and use of ITN for malaria control requirements. Two days before the distribution phase, net were convoyed to each village leader.

#### Training

In order to create equal understanding among actors at different levels of the health pyramid on the campaign implementation strategies, training was organized in a cascade manner at central, department and district levels [15]. In each department, separate micro-planning workshops and training of trainers (ToT) sessions on implementation took place to train the health zone and district coordination groups. Significant adaptations were required for the training of the distribution supervisors and teams in light of the COVID-19 pandemic and the urgency to get ITNs into households through the revised distribution strategy.

Therefore, the following precautions have been taken:

- Training was adapted to take place over three hours with a maximum of 18 people per class.
- Hygiene and safety measures were put in place (hand washing facilities, physical distancing, scanning of trainees’ badges rather than fingerprinting, health check).
- Rooms were cleaned thoroughly before and after every session.
- Content for the shortened training sessions was revised to include door-to-door distribution techniques, with the use of smartphones, and hygiene measures emphasizing the importance of keeping at least one metre physical distance from any other person.
- Audiovisual files and the electronic version of the distribution guide were shared with the distributors at the end of the training to enable them to review the content of the training once at home.
- Whatsapp groups have been created between trainers and distributors to facilitate exchanges after training.

#### Households enumeration

**Fig. 3:**
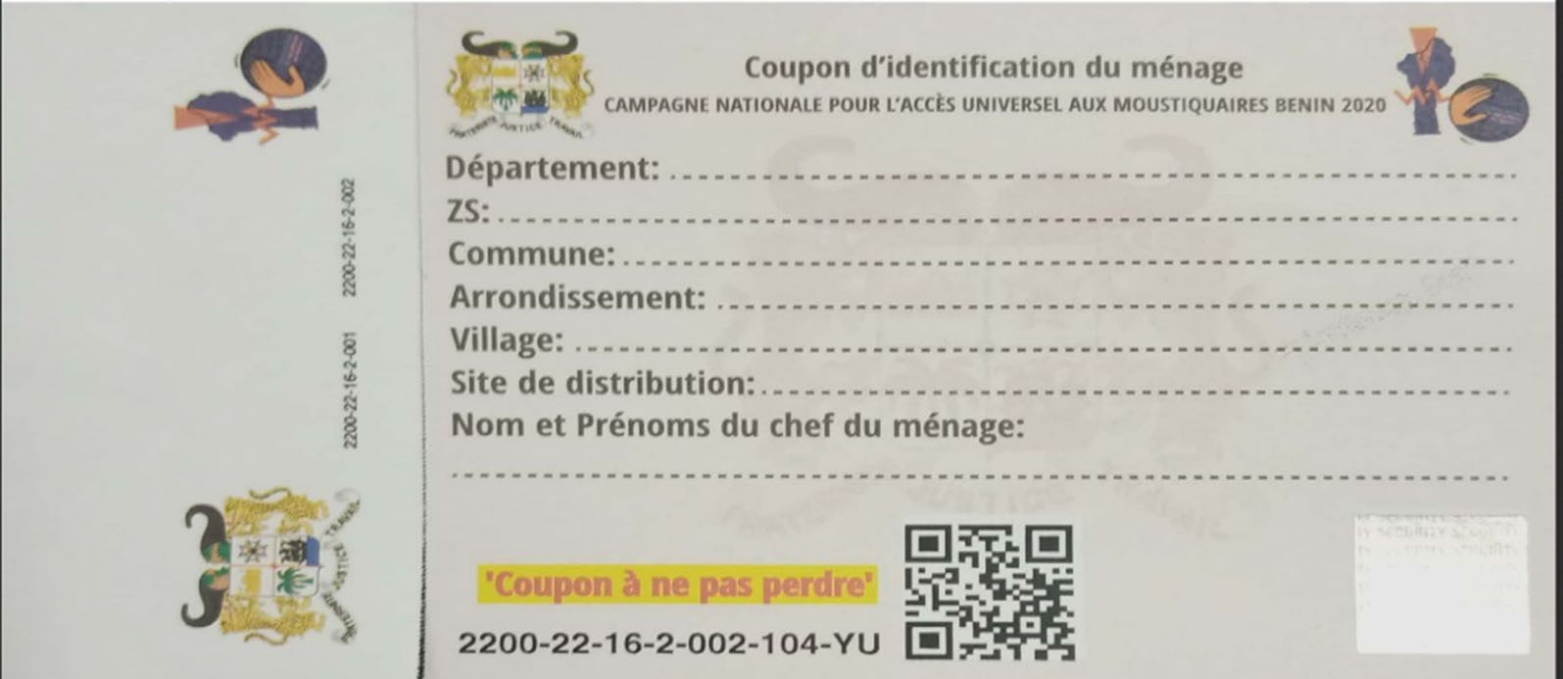
Coupon for household registration

Household enumeration was conducted by volunteers who had at least a grade 7 at secondary school. Each enumerator team was made up of two people. The first person was equipped with a smartphone to record the household’s informations. The second person delivered to the household a voucher for ITNs in the form of coupon with a Quick Response (QR) code (Fig. 3), which is the unique identifier of the household. The coupon is then later exchanged for the corresponding number of ITNs in the distribution phase during which, the coupon once scanned, generates all the household informations and displays the accurate number of nets to be redeemed, based on the applied distribution key (1–2 persons = 1 ITN; 3–4 persons = 2 ITNs; 5–6 persons = 3 ITNs; …….0.19–20 persons = 10 and more than 20 persons = 10 ITNs). The enumerator teams finally delivered key messages on malaria and the importance of sleeping under a ITN. The teams then progressed from house to house so as to cover all the households in the geographical area assigned to them, and had to register 60 households in rural areas against 70 in urban areas per day over a period of 16 days.

#### Mass distribution

The campaign was spread out in two phases. As it was the first time that Benin implemented a digital ITNs campaign, a pilot phase at the scale of a health zone was organized as a prelude to the national phase in order to understand the difficulties and constraints related to the use of digital tools.

##### Pilot phase: fixed distribution strategy

**Fig. 4:**
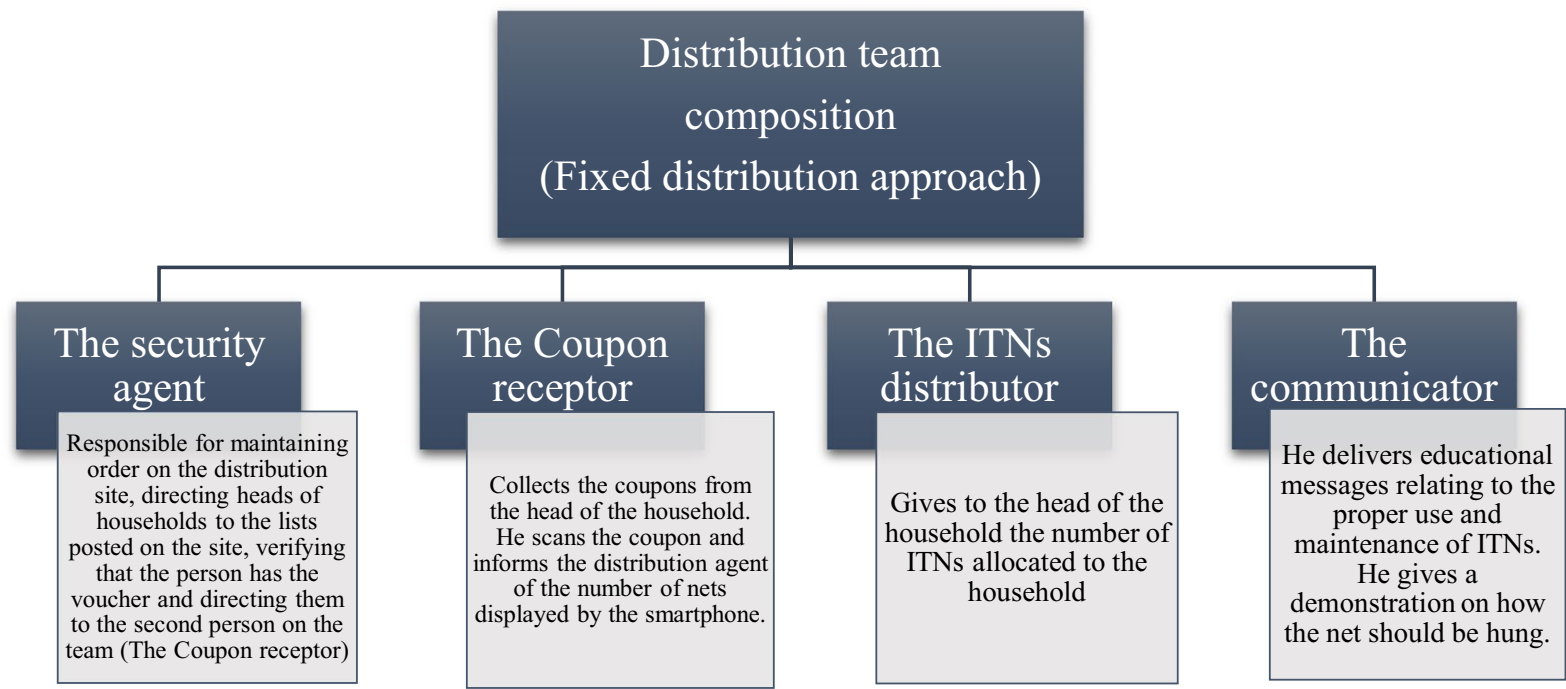
Distribution team composition and roles in fixed distribution approach

The initial approach was a fixed distribution strategy. During the pilot phase, which has implemented in one health zone (Abomey-Calavi/Sô-Ava) in the Atlantic department, ITNs distribution was done at fixed sites at the village level at a public place chosen for this purpose. Each household presented their coupon in exchange for ITNs. The number of ITNs to be allocated per household was displayed by the smartphone once the coupon has been scanned. The distribution teams involved in the distribution phase (planned for 4 continuous days, with 2 days extra) were composed of four fixed agents (Fig. 4).

##### National phase: door‑to‑door distribution

**Fig. 5:**
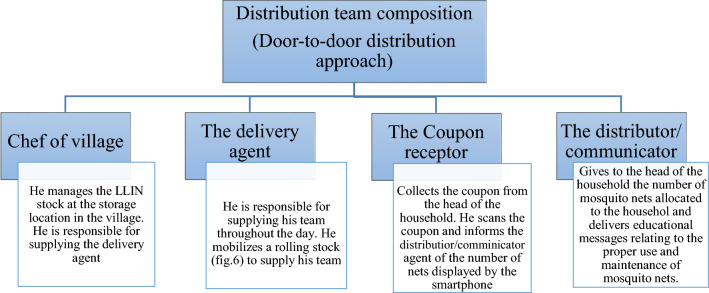
Distribution team composition and roles in door-to-door distribution approach

**Fig. 6:**
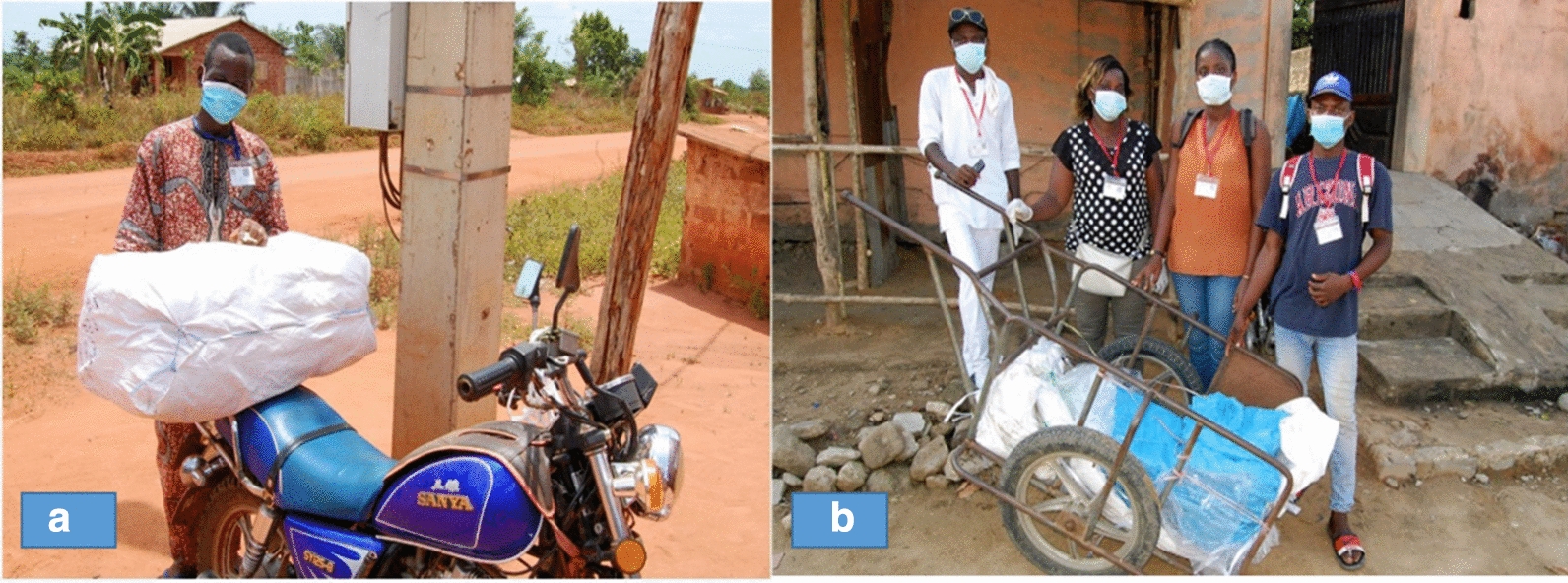
Rolling stock mobilized by the delivery agent to supply the distribution team; **a** motorbike, **b** rickshaw

During the nationwide phase, between the enumeration phase and the distribution phase, the COVID-19 outbreak occurred. Fixed distribution approach was not suited to this context. It became, therefore, necessary to revise the distribution approach. Thus, the distribution protocol was revised into a door-to-door distribution approach. By this approach, a distribution team directly delivered ITNs to recipients at their homes. The number of distribution team members remained the same, however their roles have been revised to adapt to the new distribution approach (Figs. 5, 6).

### Monitoring of household enumeration coverage

External monitoring was carried out by an external firm during the household enumeration. A rapid monitoring was carried out using Lot Quality Assurance Sampling (LQAS).

All of 77 communes were monitored. Monitoring results were shared at time with the actors and supervisors, in order to return to complete enumeration in low coverage areas.

### Supervision and coordination

At the national monitor and district supervisor levels, planned field-based activities were reduced in scale. A daily scrutiny of the distribution data uploaded from the smartphones and a virtual meeting each evening allowed supervisors and monitors to focus on problem areas and challenges that could then be addressed and resolved. At the local level, supervisors focused on ensuring that distribution teams adhered to the covid-19 safety measures, as well as ensuring planning and management of the daily team movement plans. Their responsibility included checking the health of distribution team members each day and not allowing them to continue if they showed any covid-19-like symptoms. As yet, results of the local supervision have not been thoroughly analyzed, although anecdotally, it seems that it was quite a challenge for distribution teams to adhere closely to the distancing regulations. In addition, a whatsapp group has been created at the national level, which integrates the actors at different levels in order to resolve the difficulties and situations during the distribution.

### Communication

The plan for communication included radio and television slots, town announcers and advocacy at every level. Advocacy meetings were completed in advance of the household registration phase, engaging leaders for the entirety of the campaign process. In advance of implementation of the revised strategy, messages were modified slightly to inform about the change of strategy, the new dates and the measures being taken to prevent transmission of COVID-19. As well as radio, television and town announcers, mobile messaging and audio call messages (for the less literate) were used. Community leaders were involved in local mobilization and were asked to be alert to any miscommunication that they heard about ITNs or COVID-19, and to report these to the community supervisor. Following the distribution, communication reinforced the messages passed to households by the distribution teams, i.e. proper airing of new ITNs, use of ITNs, hanging techniques and measures to prevent COVID-19.

## Results

### Household enumeration outcome

A total of 3,175,773 households were registered (more 9.3% from projection) corresponding to a total of 14,423,998 persons (13.55% more from projection) (Table 1). The real ITNs needs are, therefore, known after the enumeration. It was after the household enumeration that ITNs were conveyed to the villages.

**Table 1:** Registered population, households, and LLINs needs after household registration

Department	Household enumeration			Population enumeration		
	Registered household	Projected household	Variation (%)	Projected population	Registered population	Variation (%)
Alibori	278,720	308,208	10.58	1,114,879	1,295,882	16.24
Atacora	246,730	263,436	6.77	986,921	1,119,036	13.39
Atlantique	418,775	461,679	10.25	1,675,099	2,012,262	20.13
Borgou	385,979	444,718	15.22	1,543,914	1,887,852	22.28
Collines	230,078	238,856	3.82	920,314	984,887	7.02
Couffo	238,214	242,222	1.68	952,855	983,850	3.25
Donga	174,224	195,386	12.15	696,897	767,952	10.2
Littoral	217,978	233,769	7.24	871,913	960,950	10.21
Mono	159,037	162,903	2.43	636,149	624,841	− 1.78
Oueme	352,186	395,077	12.18	1,408,742	1,691,938	20.1
Plateau	200,406	212,236	5.9	801,624	849,786	6.01
Zou	273,446	312,668	14.34	1,093,785	1,244,762	13.8
Benin	3,175,773	3,471,158	9.3	12,703,091	14,423,998	13.55

### ITNs distribution outcome

Of the 8,609,873 ITNs procured, 7,652,166 were distributed through mass distribution to the beneficiaries. The balance from the procurement was kept to be used for routine distribution. A total of 3,240,259 households were served in the whole country, which corresponds to 93.35% of enumerated households. Moreover, 94.16% (which corresponds to a total of 13,581,637 people) of enumerated population received ITNs during the distribution phase (Table 2).

**Table 2:** Households served and distributed ITNs

Department	ITNs ditribution		Served households			Protected population		
	Allocated ITNs	Distributed ITNs	Registered households	Served households	Coverage (%)	Registered population	Projected population	Coverage (%)
Alibori	726,159	699,957	308,208	295,702	95.94	1,295,882	1,244,966	96.07
Atacora	627,123	601,891	263,436	250,969	95.27	1,119,036	1,073,365	95.92
Atlantique	1,126,189	1,022,847	461,679	412,832	89.42	2,012,262	1,828,934	90.89
Borgou	1,058,693	1,007,956	444,718	419,262	94.28	1,887,852	1,795,999	95.13
Collines	555,937	527,424	238,856	224,381	93.94	984,887	934,817	94.92
Couffo	557,840	535,059	242,222	230,662	95.23	983,850	943,350	95.88
Donga	435,622	407,922	195,386	181,645	92.97	767,952	718,628	93.58
Littoral	538,443	488,526	233,769	208,507	89.19	960,950	870,543	90.59
Mono	358,579	344,972	162,903	155,394	95.39	624,841	600,769	96.15
Oueme	947,304	891,011	395,077	368,360	93.24	1,691,938	1,589,614	93.95
Plateau	482,999	453,014	212,236	197,056	92.85	849,786	797,408	93.84
Zou	704,789	671,587	312,668	295,489	94.51	1,244,762	1,183,244	95.06
Benin	8,119,677	7,652,166	3,471,158	3,240,259	93.35	14,423,998	13,581,637	94.16

## Discussion

The 2020, ITNs mass distribution campaign in Benin had interesting features. Not only was distribution digitized for first time in Benin, but also this distribution has to be implemented in the particular context of the COVID-19 pandemic.

The benefit of digitizing the ITNs distribution campaign is obvious. It enabled accurate and efficient implementation phases of the campaign. It also improved the speed in data collection and monitoring as far as household enumeration and ITNs distribution are concerned. The use of the digital tools and dashboards enable field teams and supervisors to review the distribution data, reports and maps generated each day from any location in real time as prompt monitoring and supervision feedbacks were shared by overseeing supervisors via WhatsApp and other communication tools. The digital platform was also used to send key malaria messages in form of short messaging service (SMS) on malaria, on the use and care of the ITNs distributed to households, and to inform about the expected benefits of the nationwide distribution of ITNs.

In addition, several other key factors enabled the mass campaign to continue during the COVID-19 pandemic, and deserve to be highlighted:

- Strong support from the Government of Benin, through the Ministry of Health and the NMCP, to continue with the implementation of the ITNs campaign in advance of the high transmission malaria season, during and despite the COVID-19 pandemic.
- Effective coordination between international partners (Global Fund, World Health Organization, RBM Partnership to End Malaria, Alliance for Malaria Prevention, Bill and Melinda Gates Foundation) and the NMCP and in-country partners (the United States President’s Malaria Initiative, Catholic Relief Services)
- Regular communication between the main campaign funder (Global Fund) and the NMCP for timely decision-making to avoid delays
- Rapid problem-solving (for example, sourcing of COVID-19 protective materials for campaign workers) by the NMCP with support from the Ministry of Health and partners
- Flexibility in modifying procurement procedures to minimize delays in the campaign implementation
- Use of an electronic system for data collection that facilitated a “no touch” approach during the ITN distribution and payment of campaign workers.

The results of this campaign showed that the enumerated population was greater than the projection (13.55% more). However, since net quantification had been made with a buffer (30% security stock), this increase did not have an impact on the quantity required to ensure universal access. The door-to-door distribution of ITNs to households provided an acceptable distribution coverage rate (94.16%) and offered an opportunity for demonstrating net-hanging and face-to-face health education on ITNs use and ways of reducing net wear and tear [16]. The door-to-door delivery approach was greatly appreciated by the population. Fixed point distribution was used during the pilot phase, but this approach was not suited in the context of the COVID-19 pandemic, in terms of compliance with barrier measures. A multi-country comparison of ITN delivery strategies based on 14 surveys from five African countries did not find a significant association between delivery strategy and ownership of a net from the campaign [17–21].

Despite the satisfactory results of the 2020 ITNs campaign, some key challenges deserve to be shared:

- Change in distribution protocol: the first challenge was the need to change the distribution protocol (door-to-door approach instead of the fixed distribution previously planned) while campaign was already underway. The initial protocol was, therefore, quickly revised by a technical team.
- Challenges with COVID-19 protection materials: given the spread of the pandemic, additional materials had to be ordered with short notice and delivery time. The biggest challenge was related to the availability of personal protection equipment (PPE) in sufficient quantities on time, particularly masks.
- Challenges with payment of the people involved in the distribution (the ‘actors’). At the end of the each activity phase digital timesheets were generated from the system that was used to enable the efficient payment of staff for the number of days work as registered on the system using the unique QR code provided for each staff, to validate their presence in the field for each day’s activities. However, as payment has be done through mobile money account, incorrect phone numbers and unreported team replacements affected the on-time payment of some of the actors.
- Availably of supervision teams: the same supervision teams were sometimes involved in other response activities against COVID-19.
- Budget implications: changes to the budget were made quickly in line with the new door-to-door strategy, taking into account Benin’s specific geographic and logistical context and available human resources. Given the urgency of the COVID-19 situation and remaining funds in the country’s grant, the Global Fund was able rapidly to approve the amendments. Important modifications included the necessary increase in days for community mobilization, briefings and training, supervision and distribution as well as for purchase of PPE.

ITNs mass campaigns are a major intervention for malaria prevention and also for other arthropod-borne diseases, such as dengue, filariasis and viral infections [22, 23]. The burden imputable to malaria is heavily concentrated in sub-Saharan Africa where cases associated with COVID-19 are increasing [24]. Although COVID-19 has the potential to cause substantial disruptions to health services, due to response measures limiting usual programmatic activities, it is important to continue to prevent malaria by adapting control activities to the context of the pandemic. Recent modelling study showed that the interruption of planned ITNs campaigns due to COVID-19 pandemic could lead to a loss of life-years over 5 years that is of the same order of magnitude as the direct impact from COVID-19 in places with a high burden of malaria.

## Conclusion

Benin has implemented the digitalized mass distribution campaign of ITN in the particular context of the COVID-19 pandemic. Despite the many challenges of its implementation, the campaign was successfully implemented and contributed to increasing household ownership and population access to ITNs and, therefore, contributes to the achievement of the Global Technical Strategy for Malaria 2016–2030 goals. High political commitment, engagement and support add to the financial and technical supports from partners were essential factors for this success.

## Data Availability

The datasets analysed during the current study are available from the corresponding author on reasonable request.
